# Cellular Responses to Proteasome Inhibition: Molecular Mechanisms and Beyond

**DOI:** 10.3390/ijms20143379

**Published:** 2019-07-10

**Authors:** Nicolas Albornoz, Hianara Bustamante, Andrea Soza, Patricia Burgos

**Affiliations:** 1 Centro de Biología Celular y Biomedicina (CEBICEM), Facultad de Medicina y Ciencia, Universidad San Sebastián, Lota 2465, Santiago 7510157, Chile; 2 Instituto de Microbiología Clínica, Facultad de Medicina, Universidad Austral de Chile, Valdivia 5110566, Chile

**Keywords:** proteasome, immunoproteasome, autophagy

## Abstract

Proteasome inhibitors have been actively tested as potential anticancer drugs and in the treatment of inflammatory and autoimmune diseases. Unfortunately, cells adapt to survive in the presence of proteasome inhibitors activating a variety of cell responses that explain why these therapies have not fulfilled their expected results. In addition, all proteasome inhibitors tested and approved by the FDA have caused a variety of side effects in humans. Here, we describe the different types of proteasome complexes found within cells and the variety of regulators proteins that can modulate their activities, including those that are upregulated in the context of inflammatory processes. We also summarize the adaptive cellular responses activated during proteasome inhibition with special emphasis on the activation of the Autophagic-Lysosomal Pathway (ALP), proteaphagy, p62/SQSTM1 enriched-inclusion bodies, and proteasome biogenesis dependent on Nrf1 and Nrf2 transcription factors. Moreover, we discuss the role of IRE1 and PERK sensors in ALP activation during ER stress and the involvement of two deubiquitinases, Rpn11 and USP14, in these processes. Finally, we discuss the aspects that should be currently considered in the development of novel strategies that use proteasome activity as a therapeutic target for the treatment of human diseases.

## 1. Introduction

In cells, protein biosynthesis is constantly assisted by molecular chaperones in order to avoid accumulation of unfolded proteins and cellular stress [1,2]. The Ubiquitin-Proteasome System (UPS) is one crucial mechanism involved in the degradation of unfolded proteins that have failed quality control. The UPS serves as the major protein quality control system in the eukaryotic cells, responsible for approximately 80%–90% of the cellular protein degradation [3]. It includes most cytosolic and nuclear proteins, short and long-lived proteins, as well as unfolded proteins, all of which are finally degraded by the proteasome [4]. First, cargoes are tagged with ubiquitin (Ub) conjugates through the sequential action of three enzymatic activities E1, E2, and E3 [3,5]. Then, cargoes are recognized by Ub receptors in the proteasome, and finally, Ub conjugates are removed by the action of deubiquitinase enzymes (DUBs) [6]. After this coordination of events the substrates are finally translocated and degraded through the proteolytic chamber of the 20S proteasome [6]. Interestingly, in autoimmune and inflammatory diseases or cancer, an increase in the activity of the proteasome is found, a feature that has been directly linked with the progression of these diseases. Thus, inhibition of the proteasome is currently considered as an attractive target for therapeutic intervention with a direct impact on the UPS pathway [7,8].

## 2. Proteasome Composition

### 2.1. The 20S Proteasome: The Proteolytic Core

**Figure 1:**
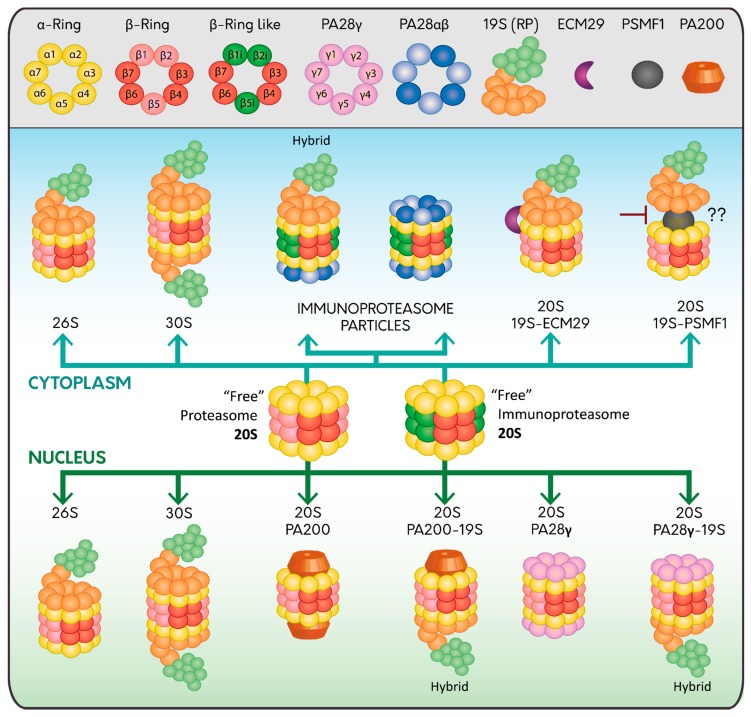
Proteasome assembly and its regulators. The constitutive 20S proteasome is assembled in α (Yellow) and β (Red) heptameric rings, constituting subunits β1, β2, β5 of the catalytic core (Light Red); meanwhile the 20S immunoproteasome catalytic core has three inducible beta subunits named β1i, β2i and β5i (Dark Green). Both types of proteasomes are found without regulators, indicated as “free” or can be associated with 19S RP (Light Orange and Light Green) either at one or both ends forming the 26S or 30S proteasome, respectively; or with the PA28 complexes either at one or both ends; PA28αβ (Blue and Light Blue; cytoplasm) or PA28γ (Pink; nucleus). Hybrid proteasome complexes (Hybrid) are also found when the catalytic core is simultaneously associated with 19S RP and another type of regulator (PA28αβ, PA28γ and PA200). Specific regulators in the cytoplasm are ECM29 (Purple) and PSMF1 (Grey), which can modify the assembly or activity of the proteasome; while in the nucleus the main proteasome regulator is PA200 (Dark Orange).

The proteasome is formed by the proteolytic core named 20S proteasome that contains the catalytic subunits needed for the proteolysis of the substrates [9,10]. The 20S proteasome has a cylinder-like structure constituted of 28 proteins arranged in four heptameric rings. The two external rings are composed of seven different α subunits (α 1–7) and the two internal rings are composed of seven different β subunits (β 1–7). The α-subunits regulate the entry of the substrate to the 20S proteasome and the interaction with the regulatory particles. The β subunits constitute the proteolytic core where β1, β2, and β5 subunits have caspase-like, trypsin-like, and chymotrypsin-like activities, respectively [11,12]. In mammalian cells, there are three inducible beta subunits named β1i, β2i and β5i, expressed in response to pro-inflammatory cytokines, especially interferon-γ during infections, cancer, inflammation, and autoimmune diseases [13,14,15]. Incorporation of these inducible subunits form a new type of 20S proteasome called the immunoproteasome, which is found predominantly, but not exclusively, in cells of the immune system specialized in major histocompatibility complex (MHC) Class I antigen presentation [16] (Figure 1). Additionally, the immunoproteasome promotes differentiation of specific populations of immune cells [17,18,19].

### 2.2. Proteasome Regulators

#### 2.2.1. PA700: The 19S Regulatory Particle (RP)

The 19S RP, also known as PA700, binds to the 20S proteasome in an ATP-dependent manner [20], either to one or both ends, forming the 26S or the 30S proteasome, respectively [21,22,23,24] (Figure 1). The 19S RP is subdivided into two sub-complexes, the base and the lid. The base is composed of 10 integral subunits. Four of these are non-ATPase subunits (Rpn1, Rpn2, Rpn10 and Rpn13) that participate in the recognition of polyubiquitylated substrates through different mechanisms. While Rpn10 and Rpn13 bind polyubiquitylated substrates directly [25,26], Rpn1 and Rpn2 dock Ub processing factors that serve as shuttles, which interact simultaneously with polyubiquitylated substrates and the proteasome [27]. Several of these shuttles have been characterized including Rad23, Dsk2, and Ddi1 [25,28,29]. The other base subunits are AAA-ATPase subunits (Rpt1-Rpt6) organized in a ring that contributes to the ATP hydrolysis needed for the unfolding, opening and entry of the substrates through the 20S proteasome [30,31,32]. On the other hand, the lid consists of nine different Rpn subunits (Rpn3, Rpn5–9, Rpn11, Rpn12, and Rpn15 (Dss1/Sem1)), which form a horseshoe-shaped structure. One key step that takes place in the lid is the deubiquitylation of the incoming substrates carried out by the structural DUB Rpn11, a highly conserved metalloprotease from yeast to human [33,34], which also helps to maintain a sufficient pool of free Ub molecules within the cells [35]. The other non-structural DUB that is transiently associated to the 19S RP is called Ubiquitin Specific Protease 14 (USP14) [36,37]. Interestingly, USP14 causes a delay in the degradation of proteasome substrates, acting as a negative regulator of the proteasome [38,39]. In agreement with these findings, selective pharmacological inhibition of USP14 enhances the proteolysis of several proteasome substrates [39,40,41].

#### 2.2.2. Proteasome Regulators: PA28αβ, PA28γ and PA200 Complexes

Two proteasome activator (PA)28 complexes composed by seven subunits have been described, the hetero-oligomer PA28αβ present preferentially in the cytosol [42], and the homo-oligomer PA28γ present only in the nucleus [43] (Figure 1). In contrast to the 19S RP, the PA28 complexes bind to the 20S proteasome (constitutively and inducibly) in an ATP-independent manner [23,44,45]. However, similar to the 19S RP, PA28 complexes bind to the 20S proteasomes either to one or both ends forming the 20S-PA28αβ or PA28αβ-20S-PA28αβ complexes. Moreover, the 20S proteasome can bind to the 19S RP and PA28αβ simultaneously forming a hybrid proteasome particle [46,47].

Interferon-γ causes the specific up-regulation of PA28αβ, but not in PA28γ [48,49,50]. High levels of PA28αβ and the immunoproteasome during inflammation explain the elevated diversity of peptides during MHC class I antigen presentation [47,51]. Interestingly, PA28αβ and the immunoproteasome have protective functions during oxidative stress conditions [52,53,54], embryonic stem cell differentiation [55] and obesity [56], indicating the relevance of their function during cellular stress scenarios.

In contrast to PA28αβ, PA28γ is more related with processes such as cell cycle [57,58], DNA repair [59,60], chromosome stability and RNA splicing [61,62], but not with antigen presentation [63,64].

Finally, similar to the PA28γ, there is another nuclear protein called PA200 that binds the 20S proteasome stimulating the hydrolysis of small peptides only, in an ATP-independent manner, process that is involved in DNA repair [65]. Additionally, it has been reported that PA200 participates in genomic stability [66,67], maintenance of glutamine homeostasis in cancer [68], and spermatogenesis [69], but its function is not fully understood.

#### 2.2.3. ECM29 and PSMF1: Two Interesting Regulators of the Proteasome Function

ECM29 is a large protein that regulates the function of the proteasome in different forms. It collaborates in the assembly and disassembly of the 26S proteasome, as the abundance of ECM29 seems to be critical for these functions [70]. ECM29 tethers the 19S RP to the 20S core, stabilizing the 20S-19S RP interaction promoting its complete assembly [70] (Figure 1). Once this step is completed, ECM29 is released and degraded by the nascent 26S proteasome [71]. In contrast, it has been shown that, under oxidative conditions, ECM29 promotes the disassembly of the 26S proteasome [72], favoring the assembly of the immunoproteasome with its regulator PA28αβ that in turns enhances the removal of oxidized proteins [72].

Other functions for ECM29 have been proposed, including its possible role in the quality control of the proteasome. It has been observed that ECM29 has preference for aberrant 20S proteasomes such as mutated versions of the proteasome or ATP-depleted proteasome particles [73]. Indeed, there is evidence that ECM29 mediates the localization of the proteasome in specific cellular compartments dependent on microtubules and motor proteins by mechanisms still not fully understood [74].

Another regulator of the proteasome is the 31 kDa protein PSMF1, also known as proteasome inhibitor (PI)31, which acts as an inhibitor of the proteasome by direct binding to the α-subunits of the 20S proteasome or by binding to the 19S regulatory particle [75,76] (Figure 1). Interestingly, the inhibitory role of the PSMF1 in the 20S proteasome can be counteracted by active p97/ valosin-containing protein (VCP), an ATPase type II protein that hijacks PSMF1 out of the proteasome [77]. In agreement with this finding, pharmacological inhibition of p97/VCP reduces proteasome activity [77]. This activity reduction could be due to the inability of p97/VCP to hijack PSMF1, or to the role of p97/VCP, in collaboration with shuttle factors, in the delivery and unfolding of polyubiquitylated substrates to the proteasome [78,79].

#### 2.2.4. Regulation of Proteasome Assembly and Function by E3 Ligases

Cells contain approximately one thousand E3 ligases that explain the high degree of substrate specificity. E3 ligases are divided into three subclasses based on their structural and biochemical features: Homologous to E6-AP carboxy terminus (HECT), really interesting new gene (RING) fingers, and U-box domains. The RING finger subclass is subdivided into two subfamilies: Cullin-containing RING-finger ligases (CRLs) and those in which the RING-finger and substrate binding domains are contained on the same polypeptide. Regardless of the well-known role of E3 ligases on proteasome substrates, it is also accepted that they have a role in the ubiquitylation of a variety of proteasome subunits [80,81,82,83]. For instance, the E3 RING ligase Not4 triggers direct ubiquitylation of the Rpt5 subunit, playing a crucial function in 26S proteasome assembly [84]. In agreement with this study, it was previously shown that deletion of Not4 was correlated with instability of the 26S proteasome leading to dissociation of the 20S proteasome and 19S RP, accumulation of polyubiquitylated proteins and a decrease in the levels of free Ub [85]. Some of these effects were explained by the finding that Not4 interacts with ECM29, a protein involved in 26S proteasome stabilization [85]. Authors described that, in the absence of Not4, ECM29 was no longer associated to the proteasome which in part explained the proteasome instability [85]. In addition to Not4, it has been shown that the E3 U-box ligase SNEV (senescence evasion factor) is also mediating the efficiency of proteasome function by the delivery of ubiquitylated substrates to the proteasome via direct binding to β7 subunit on the 20S proteasome [86]. Finally, the E3 HECT ligase UBE3A, is an enzyme implicated in the ubiquitylation of a variety of proteasome subunits, post-translational modifications that have a positive impact in proteasome activity [87]. In this regard, it has been shown that catalytic defective mutations in UBE3A, are associated with the human Angelman syndrome, resulting in an overall inhibitory effect on the proteolytic activity of the proteasome [88]. Although several findings indicate that ubiquitylation of proteasome subunits plays a positive role on proteasome function, there is still a controversy about this conclusion [80].

## 3. Pharmacological Inhibitors of the Proteasome Function

### 3.1. 20S Proteasome Inhibitors

**Figure 2:**
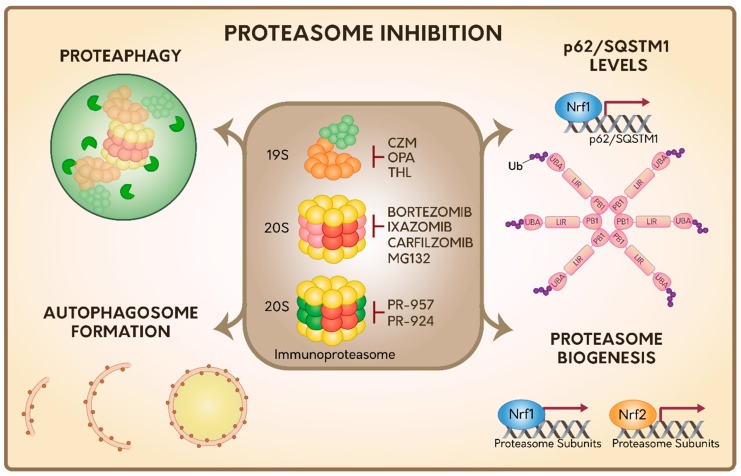
Summary of the cellular responses during proteasome inhibition. Several proteasome inhibitors have been described targeting 19S RP, 20S proteasome and immunoproteasome. In response to proteasome inhibition, several responses are activated specifically related with the 20S proteasome, including the Autophagic-Lysosomal Pathway (ALP), proteaphagy, the transcriptional upregulation of the autophagy Ub receptor p62/SQSTM1, and proteasome genes, by Nrf1 and Nrf1/Nrf2 transcription factors, respectively. The activation of the autophagy Ub receptor p62/SQSTM1 is dependent on specific post-translational modifications based on ubiquitylation. However, it is still unknown which responses could be activated under the inhibition of 19S RP or immunoproteasome.

Several reversible and irreversible proteasome inhibitors have been described to date, which in general target the active sites of the 20S proteolytic core. Among these, we found the peptide aldehydes (ALLN, MG132 and MG115), peptide boronates (PS-341 or bortezomib), epoxyketones (carfilzomib and epoxomicin), and a metabolite isolated from *Streptomyces* called lactacystin [89] (Figure 2). As many of these inhibitors negatively impact cell survival and proliferation processes they are currently considered excellent triggers of cell death and they are an attractive niche for the treatment of cancer [90,91,92,93] and inflammatory diseases [94,95,96,97,98].

One of the most widely used proteasome inhibitors in basic research is MG132. This inhibitor binds to the β5 subunit of the 20S proteasome in a reversible manner in the low ŋM range. However, in high concentrations (low µM range), MG132 can also bind to the β1 and β2 subunits of the 20S proteasome [99]. Moreover, it can also inhibit other proteases including calpains and cathepsins in high concentrations [100]. Regardless of these features, MG132 is still widely used in in vitro studies but with the appropriate controls [99]. MG132 is capable of inducing apoptosis in tumor cells [101], however, and similar to what happens in the presence of other proteasome inhibitors, tumor cells can adapt and live in the presence of this type of inhibitors through the activation of a series of mechanisms that we discuss further.

Currently only three proteasome inhibitors have been approved by the Food and Drug Administration (FDA). The first one was bortezomib approved in 2003 for the treatment of multiple myeloma and mantle cell lymphoma [102]. Bortezomib binds to β5 and β5i subunits and to a lesser extent to β2 and β1 subunits causing the inhibition of the 20S proteasome, as well as the immunoproteasome, in a slightly reversible manner [103,104]. The second one is carfilzomib, a proteasome inhibitor with improved properties compared to bortezomib [105], which was approved by the FDA in 2012 [106]. Carfilzomib binds to the catalytic β5 and β5i subunits but with a greater affinity than bortezomib and in an irreversible manner (IC_50_ values of less than 10 nM) [107,108]. The irreversible mechanism of carfilzomib allows a sustained and durable inhibition of the proteasome, a characteristic that improves its efficacy as a therapeutic agent [109]. The third one is ixazomib, the first oral proteasome inhibitor approved by the FDA in 2015. In low doses ixazomib binds specifically to the β5 subunit of the 20S proteasome and in a reversible manner. However, in high doses it can also bind to β1 and β2 subunits causing the massive accumulation of intracellular ubiquitylated proteins [110]. In comparison to bortezomib, it is thought that ixazomib offers an improvement in terms of its pharmacokinetic and pharmacodynamic profiles, however its therapeutic advantages have not been fully investigated yet in randomized clinical trials including bortezomib or carfilzomib [111].

Importantly, all FDA approved proteasome inhibitors present several side-effects in patients, including diarrhea, hematologic toxicities, peripheral neurophaties and fatigue, among other symptoms [112]. This highlights the need of better therapies including the clinical development of combinatory drugs to avoid these side-effects. In addition to this, it is known that the current inhibitors have a limited effect on solid tumors, indicating the importance of new drug discovery.

One promising drug is marizomib, a new proteasome inhibitor derived from a marine *Actinomyces*, which inhibits all three catalytic subunits of the 20S proteasome in an irreversible manner, both in vitro and in vivo [113,114]. Marizomib is currently under clinical trials showing good safety and efficacy profiles in multiple myeloma compared to other similar inhibitors [115]. Therefore, marizomib is considered a promising therapeutic strategy to be used in the treatment of solid tumors.

### 3.2. Immunoproteasome Inhibitors

Immunoproteasome is highly expressed during the course of inflammation and in different types of cancer [116,117,118]. Therefore, the immunoproteasome is currently the focus of several studies in biomedicine. In recent years, the upregulation of the immunoproteasome has been reported in a number of inflammatory and autoimmune diseases, such as ulcerative colitis [119], chronic active hepatitis [120], inflammatory bowel disease (IBD) [121,122], and Crohn’s disease [123]. In this regard, it has been shown that pharmacological inhibition of the immunoproteasome prevents lupus- and rheumatoid arthritis-like diseases, experimental colitis and colitis-associated cancer, Hashimoto’s thyroiditis, acute myocarditis, microglial activation following central nervous system injury, and allograft rejection in mouse models [19]. These findings strongly indicate that selective inhibition of the immunoproteasome is an attractive pharmacological strategy for the treatment of these diseases.

To date, most studies on immunoproteasome inhibitors have been on PR-957 [124] and PR-924 [125,126], which bind selectively and irreversibly to the β5i subunit of the immunoproteasome [127] (Figure 2). Moreover, and in contrast to bortezomib and carfilzomib, PR-957 only inhibits the immunoproteasome, without showing any effect on the 20S constitutive proteasome [124,127]. PR-924 is 100-fold more selective for β5i than to β5 compared to bortezomib and carfilzomib, which can target other activities as well [125].

This high selectivity opens up very promising treatments in the future that should reduce side effects in humans [128].

### 3.3. Inhibitors of the DUB Rpn11 in the 19S RP

Capzimin (CZM) is the first-in-class inhibitor of 19S RP proteasome, which acts inhibiting the DUB Rpn11 of the 19S RP described above [129] (Figure 2). Rpn11 removes the polyubiquitin chains of the substrates prior to their entry and degradation in the 20S proteasome, also allowing the recycling of Ub molecules. Interestingly, CZM has been proven to be effective in several cancer cell lines, including those that are resistant to bortezomib. It triggers the inhibition of cell proliferation and the induction of ER stress and cell death [129]. Likewise, other two Rpn11 inhibitors have been reported [130,131]. For instance, O-phenanthroline (OPA), which triggers ER stress and cell death in multiple myeloma cells [130] and thiolutin (THL), an antibiotic compound derivate from *Streptomyces* [131] (Figure 2). All these inhibitors have a common phenotype in mammalian cells, which is the increase in the levels of polyubiquitylated proteins, confirming its key role in the clearance of proteasome substrates. In addition, recent findings indicate that levels of Rpn11 (mRNA and protein) are increased in a variety of tumor cell types [130,132]. Thus, downregulation or inhibition of Rpn11 is currently proposed as an attractive strategy against tumor growth. Whether these inhibitors could also be a good strategy in the treatment of inflammatory and autoimmune diseases has not yet been explored.

## 4. Cellular Responses upon Proteasome Inhibition

### 4.1. Proteasome Inhibition Leads to Autophagic-Lysosomal Pathway Activation

In addition to the UPS function, the Autophagic-Lysosomal Pathway (ALP) is responsible for approximately 10%–20% of the protein degradation [3], a mechanism mostly specialized in bulk degradation of substrates including protein aggregates or organelles in lysosomes such as mitochondria, endoplasmic reticulum, lipid droplets, among others [133]. This pathway is characterized by the biogenesis of an organelle enclosed by a double lipid bilayer named autophagosome, able to engulf the cytoplasmic constituents to later be fused with lysosomes, to form a hybrid organelle called autolysosome that mediates the degradation of the cargoes. The UPS and ALP regulate the cellular proteostasis, constituting a single network to achieve protein balance [134]. Even though these mechanisms have been studied separately, growing evidence indicates that they operate in an intimate cooperation [135] supported by the fact that UPS impairment is a strong trigger of ALP activation [136,137,138,139,140]. Recently, we reviewed the interplay between UPS and ALP with a focus on the molecular players that mediate their functional crosstalk, highlighting the Ub signal as the unifying factor in cellular proteostasis maintenance [141].

The experimental approaches to block proteasome function are frequently based on the use of pharmacological proteasome inhibitors that normally target the catalytic subunits in the 20S proteasome [136,137,138,139,140,142,143,144,145], overexpression of exogenous proteins that are prone to aggregate [138,144,146,147,148,149,150], and more recently the use of genetic tools to modify the gene expression of specific subunits in the proteasome [144,150].

Early evidences showed that pharmacological proteasome inhibitors such as lactacystin and MG115 were potent inducers of ALP in neuronal cells. This was evidenced by the increase in autophagosome structures in ultrastructural studies and in positive structures with cathepsin D and ubiquitylated proteins [136,137], a phenotype that was observed even under low dose exposure, but in a chronic form [136]. In later studies, it was demonstrated that treatment with several proteasome inhibitors such as ALLN [138], MG132 [138,139,140], and bortezomib [139,143,144,145] were able to increase LC3-II levels in different cell types. LC3-II corresponds to the lipidated form of LC3 (also named Microtubule-associated protein light chain 3 (MAP1LC3)), corresponding to the orthologous protein ATG8 in yeast. LC3-II/ATG8 is a protein covalently conjugated to phosphatidylethanolamine (PE) on autophagosomal membranes, a classical marker of autophagosomes [151,152]. Moreover, bortezomib upregulates the mRNA levels of LC3 [143] and of ATG5 and ATG7 [144], two key autophagy proteins involved in the LC3 lipidation. In addition, it was reported that proteasome inhibitors favor the activation of the transcription factor EB (TFEB), promoting its nuclear shuttling [153] together with an increase in its stability [154]. TFEB is currently considered as a master regulator of the lysosomal biogenesis and autophagy, because it coordinates the expression of the CLEAR network (Coordinated Lysosomal Expression and Regulation), which is composed of at least 471 targets, including a battery of many lysosomal and ATG genes [155,156,157].

On the other hand, overexpression of exogenous proteins prone to aggregate such as the ectopic expression of Huntingtin (Htt) with polyQ repeats (polyQ-Htt) [146] or the mutant superoxide dismutase 1 (SOD1) [147] resulted in the impairment of the proteasome function. This is due to allosteric inhibition of the 20S proteasome by the oligomers, which in many cases form inclusion bodies, which act as a naturally occurring proteasome inhibition process. Interestingly, polyQ-Htt aggregates induce autophagosome formation demonstrated by conversion of LC3-I to LC3-II [149], increase in the number of LC3-positive structures [138,149] and upregulation of ALP genes [148,149]. Likewise, mutant SOD1 enhanced the conversion of LC3-I to LC3-II in neuronal cells [158]. In agreement with these findings, classical pharmacological inhibitors of ALP cause the accumulation of PolyQ-Htt [148,149,159] and mutant SOD1 aggregates [160]. All these together suggest that ALP is activated as an attempt by the cells to reduce the toxicity of these mutant proteins.

Moreover, inhibition of the 20S proteasome by the silencing of its β5 subunit or triple silencing of β1, β2, and β5 subunits in its proteolytic core, lead to the upregulation in ATG5 and ATG7 mRNA levels, key genes implicated in the lipidation of LC3-II [144]. In addition, depletion of Rpn10 and Rpn13, two of the receptors that participate in the recognition of polyubiquitylated substrates in the 19S RP cause strong activation of ALP, explaining how these cells survive the lack of these key proteins [150].

Together, this indicates that inhibition of the proteasome activity either by pharmacological inhibitors, protein aggregates or genetic tools designed to modify the expression of specific subunits of the proteasome, lead to the activation of the ALP system as a response to maintain the cellular proteostasis (Figure 2).

### 4.2. Inclusion Body Formation and Associated-Proteins under Proteasome Inhibition

Under treatment with proteasome inhibitors several proteins are accumulated forming protein aggregates. Once formed, aggregates accumulate forming inclusion bodies [161], which have been proposed could end up triggering side-effects during long term proteasome inhibition similar to the ones observed with the expression of pathogenic mutant proteins [162,163].

Different types of inclusion bodies have been identified in mammalian cells including the aggresomes [161] and stress granules (SGs) [164]. Initially, it was believed that protein aggregates were diffusing within cells, however it is currently accepted that the aggresome is the result of a coordinated retrograde transport of aggregates through microtubules in a dynein-dependent manner [161,165,166]. Aggresomes are specifically located at the perinuclear region near to the microtubule-organizing center (MTOC), where they seem to play a protective role when cells face proteotoxic stress by proteasomal inhibition. Aggresome formation is coordinated with the transport of autophagosomes and lysosomes to the same location to facilitate protein clearance [137,138]. On the other hand, it has been demonstrated that SGs are formed in cancer cells in response to bortezomib treatment, where they seem to promote tumor cell survival due to an inhibition in the degradation of mRNAs that encode key survival proteins [167]. Interestingly, these two types of inclusion bodies have been connected with p62/Sequestosome 1 (SQSTM1), a protein that colocalizes with both structures [168,169,170].

p62/SQSTM1 was the first mammalian autophagic Ubreceptor discovered [171,172,173]. It consists of a multi-domain protein with several protein-protein interaction motifs, which are key in its function. Its Ub-associated domain (UBA) interacts with the Ub-chains attached to the substrates [171,174], its Phox and Bem1 domain 1 (PB1) domain play a crucial role in its oligomerization and in its function as an Ub receptor [172,175]. Its LC3-interacting region (LIR) domain mediates binding to LC3 and Gamma-aminobutyric acid receptor-associated protein (GABARAP) subfamily proteins, which is a crucial step for its association to autophagosome membrane and delivery of the cargo for degradation [133,172,173,176] (Figure 2).

Interestingly, several lines of evidence indicate that p62/SQSTM1 is able to sense proteasome inhibition. In particular, mRNA and protein levels of p62/SQSTM1 are upregulated upon expression of polyQ Htt [177], which impairs proteasome activity, as we mentioned before [146]. Similar results are found on pharmacological inhibition of the proteasome in *Drosophila* [178] and in mammalian cells [145,179,180,181,182,183]. In addition, it was found that bortezomib was able to increase a rapid and selective up-regulation of p62/SQSTM1 through the transcription factor Nuclear respiratory factor 1 (Nrf1) involved in *de novo* synthesis of the proteasome [183,184] (Figure 2). Moreover, these authors found that after a prolonged exposure to proteasome inhibitors, several ATG genes and autophagy Ub receptors were upregulated, which explains the activation of ALP pathway upon these treatments [183]. Interestingly, the rapid induction in the expression of p62/SQSTM1 upon proteasomal inhibition seems to be independent of other transcription factors previously reported such as Forkhead box O3 (FoxO3), phospho- Eukaryotic Initiation Factor 2 p-(eIF2α), Nuclear factor erythroid 2 (Nrf2), NF-κB: Nuclear Factor kappa B (NF-κB), and TFEB [183]. Finally, recent reports have shown that proteasome inhibition by MG132 increases the ubiquitylation state in p62/SQSTM1 [182]. In a similar manner, bortezomib increases the ubiquitylation of p62/SQSTM1 in the K420 residue, located within its UBA domain, increasing its function as an autophagy Ub receptor [145] (Figure 2). These findings strongly suggest that post-translational modifications (PTMs) in p62/SQSTM1 could act as another powerful attractive strategy that mediates the interplay between proteasome inhibition and ALP activation.

### 4.3. Proteasome Inhibition Induces Proteaphagy

The abundance and quality of the proteasome is finely regulated by the recent discovery of a process called proteaphagy, a conserved mechanism from plants to humans [185,186,187]. Proteaphagy is the process by which inactive and aged proteasomes are eliminated within cells through ALP [188]. Initially, it was found that the 20S proteasome accumulates within lysosomes in rat liver cells when they were treated with nutrient starvation or leupeptin, a strong inhibitor of lysosome proteases [189]. In addition to this report, a proteomic study revealed the presence of 26S proteasome subunits in autophagosome enriched fractions [190]. Therefore, these findings gave the first idea that the 20S and 26S proteasome could be degraded by ALP (Figure 2).

Studies in *Arabidopsis thaliana* gave the first confirmation about this hypothesis and named this mechanism as proteaphagy [186]. Activation of ALP in *Arabidopsis* with nitrogen deprivation or MG132 showed the proteasome in autophagic bodies decorated with ATG8 [191]. Moreover, it was found that proteaphagy induced by MG132 is a process dependent on Rpn10 [185]. Rpn10 is found as an intrinsic Ub receptor of the 19S RP, or as a cytosolic protein within cells [32,192,193]. The cytosolic pool can simultaneously bind both, ATG8 and Ub, acting as a selective autophagy receptor that targets inactive and ubiquitylated 26S proteasomes to autophagosomes [185]. Interestingly, levels of Rpn10 increased by MG132 treatment, probably due to its strong association to the 26S proteasome, a result that was reproduced with the inhibition of the proteasome by bacterial infection [194]. All this suggests that proteaphagy is activated to eliminate inactive proteasome, which could help to reduce a toxic scenario during cellular stress.

Later studies in yeast demonstrated that MG132 could also activate proteaphagy, but using a different autophagy Ub receptor named Cue5 [186]. In addition, nitrogen deprivation also induced-proteaphagy in yeast, but few aspects of this pathway are currently known [195,196].

Proteaphagy in mammalian cells has been recently discovered [187], as a process dependent on the autophagy Ub receptor p62/SQSTM1 during starvation, which mediates the recognition of the ubiquitylated proteasome subunits [187]. To date, nothing yet is known about the role of proteaphagy under MG132 treatment in mammalian cells. Considering the fast and specific upregulation of p62/SQSTM1 upon proteasome inhibition, it is a good candidate to be explored.

### 4.4. Proteasome Inhibition Induces Proteasome Biogenesis Dependent on Nrf1 and Nrf2 Transcription Factors

Each time the proteasome function is inhibited, cells respond activating *de novo* proteasome synthesis through the upregulation in the expression of specific genes, encoding subunits of the 20S proteasome or the 19S RP. This proteasome recovery mechanism is highly conserved between *Drosophila* [178] and mammalian cells [91,197] and relies on the function of the transcription factor Nrf1 [184] (Figure 2). Nrf1 is an endoplasmic reticulum (ER) transmembrane protein that, under normal conditions, is subjected to ER-associated degradation (ERAD) [198]. Proteasome inhibition stabilizes Nrf1 on the ER membrane, where it gets cleaved by the DNA Damage Inducible 1 Homolog 2 (DDI-2) protease, resulting in the release of a proteolytic fragment of Nrf1 from the ER into the nucleus activating the transcription of proteasome subunit genes [199]. Thus, activation of Nrf1 in response to proteasome inhibition, constitutes a mechanism of resistance in cancer treatment [200]. Recently, it was reported that Nrf1 stability is also regulated by O-linked N-acetylglucosamine (O-GlcNAc) modifications in serine or threonine residues by the O-linked N-acetylglucosamine transferase (OGT) [201], an enzyme that is highly active in many types of cancer [202]. Interestingly, nuclear Nrf1 interacts with Beta-Transducin Repeat Containing E3 ligase (β-TrCP), an E3 responsible for its ubiquitylation and proteasome degradation, which in turn leads to the reduction in *de novo* proteasome synthesis [203,204]. Interestingly, when Nrf1 is modified by O-GlcNAc modifications, it is unable to bind to β-TrCP, which explains why high OGT activity correlates with high levels of both, Nrf1 and proteasome [201].

In addition, the Nrf2 transcription factor, which is found in very low levels under normal conditions due to its rapid degradation through the proteasome is highly upregulated under oxidative stress and other stress conditions [205]. Once in the nucleus, Nrf2 promotes the synthesis of several proteins with antioxidant properties through common elements of response named Antioxidant Responsive Elements (ARE), found in genes such as the glutamate cysteine ligase catalytic subunit (GCLC), glutathione peroxidase (GPX-1), heme oxygenase-1 (HO-1), and catalase [206,207]. Moreover, Nrf2 has also been implicated in xenobiotic stress, mitochondrial respiration, stem cell quiescence, mRNA translation, autophagy and unfolded protein response (UPR) [208,209,210,211,212,213]. Importantly, Nrf2 cooperates in the expression of 20S proteasome and PA28αβ subunits helping to restore oxidative damage and cell viability [214,215,216] (Figure 2). In contrast, expression of the immunoproteasome is regulated by TNF-α and IFN-γ in a mechanism totally independent of Nrf1 and Nrf2 [217]. Hence, different types of proteasome are differentially expressed in cells according to cellular conditions and functions.

Together these findings indicate that proteasome inhibitors by themselves are not a good strategy for treatment, highlighting the importance of combined therapies that incorporate the role of newly-synthesized proteasome and its assembly. Blocking *de novo* proteasome synthesis by the regulation of Nrf1 stability, or by affecting Nrf2/ARE have recently been proposed as an interesting alternative strategy [218].

## 5. Role of UPR and ER Stress in ALP Activation During Proteasome Inhibition

Several studies have indicated that proteasome disruption either by pharmacological inhibitors [139,143] or by proteins prone to aggregate [149,219] leads to ER stress. Proteasome collaborates actively in ER quality control through the proteolysis of unfolded proteins in the lumen of the ER, acting as an important player in ERAD. Briefly, sophisticated machinery selects unfolded proteins in the lumen of the ER, by the recognition of specific branching of sugars that deliver these proteins back to the cytosol by retrotranslocation. In the cytosol, retrotranslocated proteins are polyubiquitylated and extracted from the ER membrane, where they are finally targeted to the proteasome for degradation [220]. Thus, proteasome inhibition increases the levels of unfolded proteins in the ER, a condition referred to as ER stress.

Cells deal with the accumulation of unfolded proteins with the activation of the UPR, involving the induction of molecular chaperones, translation attenuation, and upregulation of ERAD activity, all to avoid cell death [221]. Three different ER stress transducers in mammalian cells have been described called, Protein kinase R like endoplasmic reticulum kinase (PERK), Inositol-requiring enzyme 1 (IRE1), and Activating Transcription Factor 6 (ATF6), which sense the presence of unfolded proteins in the ER lumen and transduce signals to the cytoplasm and the nucleus [222,223]. PERK activation leads to the phosphorylation of the α subunit of the translation initiation factor, eIF2α, which inhibits the assembly of the 80S ribosome and inhibits the global protein synthesis [224,225], providing time to cells to implement the reprogramming of the transcriptome. Activation of IRE1 and ATF6 promotes the transcription of UPR target genes. IRE1 is an ER resident transmembrane protein with endoribonuclease/kinase activity, which facilitates the cytoplasmic splicing of X-box binding protein 1 (XBP1) mRNA, generating the active form of the XBP1 transcription factor, named XBP1s (spliced) [226,227]. XBP1s enhances the expression of UPR genes involved in protein quality control, disulfide linkage and ERAD pathway components [226,228,229]. ATF6 is a membrane-bound transcription factor localized in the ER that serves as a sensor of ER stress as well as a transcriptional activator of UPR target genes. ATF6 undergoes proteolytic cleavage in response to ER stress, whereas its N-terminal fragment is translocated to the nucleus and increases the expression of a network of genes, including ER chaperones such as binding-immunoglobulin protein (BiP)/Glucose-Regulated Protein, 78 kDa (GRP78), and ERAD pathway components [230,231].

Interestingly, it is known that autophagosome formation is accelerated in the cells under ER stress [232]. In fact, early studies demonstrated that the only UPR signaling responsible for autophagosome formation under ER stress was IRE1 [232]. Moreover, the authors demonstrated that, in IRE1-deficient cells, the activation in the induction of autophagosome structures by nutrient starvation was perfectly normal, suggesting a specific mechanism for ALP induction during ER stress. In agreement with these findings, activation of ALP by MG132 and bortezomib was suppressed upon knock down or gene deletion of IRE1 [139]. However, other findings with protein aggregates as triggers of ER stress have contradicted this conclusion. For instance, activation of ALP with the ectopic expression of polyQ-Htt is abolished by PERK signaling pathway disruption [149]. Similar results have been observed with the expression of a protein prone to aggregate in ER Dysferlin L1341P [233] or in cells expressing eIF2α mutant in the PERK phosphorylation site, treated with the proteasomal inhibitors bortezomib and NPI-0052 [144].

Together, this indicates that UPR signaling pathways, specifically IRE1 and PERK are involved in ALP activation during ER stress. However, how these signaling pathways mediate ALP activation is not well understood. New insights were recently found through a microarray analysis in the presence of tunicamycin, but not with proteasome inhibitors. The authors confirmed that IRE1 and PERK were required for the upregulation of transcripts of several autophagy related genes, including the autophagy Ub receptors p62/SQSTM1, Neighbor of BRCA1 gene 1 (NBR1), and BCL2 Interacting Protein 3 Like (BNIP3L)/ NIP-3-Like Protein X (NIX), that upon tunicamycin treatment [234], delivered cargoes to the autophagosomes by binding with LC3 through the LIR motif [235]. Consistent with these findings, it was previously found that treatment of neuronal cells with MG132, lactacystin or PSI increased the transcript of p62/SQSTM1 [179] as well as its homologue in flies [236].

Altogether, in vitro and in vivo studies indicate that proteasome inhibition activates ALP by a mechanism that involves IRE1 and PERK signaling pathways, highlighting the crucial role of ER in autophagosome formation. However, further systematic studies oriented at clarifying the link between proteasome inhibition, UPR signaling, and ALP activation are needed, considering the variety of proteasome particles and their regulators.

## 6. Role of DUBs in ALP Activation During Proteasome Inhibition

As was mentioned previously, an important regulatory step for efficient degradation through the 20S proteasome is the deubiquityation of the substrates. However, in addition to this function, it has been proposed that USP14 and Rpn11 contribute to the interplay between UPS and ALP. USP14 activity regulates ALP because it mediates the deubiquitylation of Beclin1, a key step that promotes its binding to its partners ATG14L and UVRAG enhancing ALP function under normal scenarios [237]. When USP14 function is pharmacologically inhibited, proteasome activity is highly activated [39,40,41]. Interestingly, inhibition of USP14 also has an impact on ALP function dependent on the cell type. In H4 neuroglioma cells, USP14 inhibition causes an upregulation of autophagy [237]. In contrast, in Hek-293 the opposite phenotype is observed [41]. Regardless of this difference, it highlights a close interplay between UPS and ALP mediated by USP14 activity.

Another interesting DUB implicated in this interplay is Rpn11. Under inhibition of the proteasome, Rpn11 cleaves the ubiquitylated substrates in a non-canonical manner releasing free K63-Ub chains to the cytosol [238]. Interestingly, it was proposed that free cytosolic K63-Ub chains activate the clearance of protein aggregates by ALP dependent on Histone deacetylase 6 (HDAC6) [239,240]. K63-Ub chains trigger the interaction of HDAC6 with dynein motors allowing the efficient transport of aggregates along the microtubules to the MTOC. This transport seems to be critical to relocate these aggregates in the aggresome at the perinuclear region, where ALP takes place [241]. Moreover, binding of K63-Ub chains to HDAC6 favors the deacetylation of the cytoplasmic protein cortactin, which facilitates the assembly of the F-actin network, which is also necessary for ALP function [242,243].

## 7. Concluding Remarks and Perspectives

We have summarized the different types of proteasome particles present in mammalian cells, describing the most common regulatory mechanisms for proteasome regulation and the cellular responses upon proteasome inhibition (Figure 2). Due to the several side effects described for proteasome inhibitors, it is important to search for new drugs with different specific targets. One interesting drug is MLN4924, currently in phase I of clinical trials, an inhibitor of the CRLs subfamily of E3 RING ligases that abolishes the degradation of 20% of all cellular proteins and that has showed few side effects in mice. This strategy could also be extended in the future to the development of novel RING/HECT/U-box E3 ligases inhibitors with special focus on Not4, SNEV, and UBE3A, E3 ligases directly implicated in the control of proteasome assembly and function [244,245]. Finally, the crucial role of ER stress and UPR signaling pathways and the role of DUBs in ALP activation are highlighted as the major players that contribute to cellular adaptation upon proteasome inhibition.

## Abbreviations

ARE	Antioxidant responsive elements
ALP	Autophagic-Lysosomal Pathway
ATF6	Activating Transcription Factor 6
ATG	Autophagy related protein
Bip	Binding-Immunoglobulin Protein
BNIP3L	BCL2 Interacting Protein 3 Like
β-TrCP	Beta-Transducin Repeat Containing E3 Ubiquitin Protein Ligase
CZM	Capzimin
CLEAR	Coordinated Lysosomal Expression and Regulation
CRLs	Cullin-containing RING-finger ligases
CUE5	Coupling of ubiquitin to ER degradation
DDI-2	DNA Damage Inducible 1 Homolog 2
DUBs	Deubiquitinase enzymes
EIF2α	Eukaryotic Initiation Factor 2 alpha
ER	Endoplasmic Reticulum
ERAD	ER-associated degradation
FDA	Food and Drug Administration
FoxO3	Forkhead box O3
GABARAP	Gamma-aminobutyric acid receptor-associated protein
GCLC	Glutamate cysteine ligase catalytic subunit
GPX-1	Glutathione peroxidase 1
GRP-78	Glucose-Regulated Protein, 78kDa
HECT	Homologous to E6-AP carboxy terminus
HDAC6	Histone deacetylase 6
HO-1	Heme oxygenase-1
Htt	Huntingtin
IBD	Inflammatory bowel disease
IRE1	Inositol-requiring enzyme 1
LIR	LC3-interacting region
MHC	Major histocompatibility complex
MAPLC3	Microtubule-associated protein light chain 3
MTOC	Microtubule-organizing center
NBR1	Neighbor of BRCA1 gene
NF-κB	Nuclear Factor kappa B
NIX	NIP-3-Like Protein X
Nrf1	Nuclear respiratory factor 1
Nrf2	Nuclear factor erythroid 2
O-GlcNAc	O-linked N-acetylglucosamine
OGT	O-linked N-acetylglucosamine transferase
OPA	O-phenanthroline
PA	Proteasome activator
PB1	Phox and Bem1 domain
PE	Phosphatidylethanolamine
PERK	Protein kinase R like endoplasmic reticulum kinase
PI	Proteasome inhibitor
PolyQ-Htt	Htt with polyQ repeats
PTMs	Post-translational modifications
RING	Really interesting new gene
RP	Regulatory Particle
Rpn	Regulatory particle of non-ATPase subunit
Rpt	Regulatory particle of triple-A subunit
SG	Stress granules
SQSTM1	Sequestosome
SOD1	Superoxide dismutase 1
TFEB	Transcription factor EB
THL	Thiolutin
Ub	Ubiquitin
UBA	Ub-associated domain
UPS	Ubiquitin-Proteasome System
USP14	Ubiquitin specific protease 14
UVRAG	UV Radiation Resistance-Associated Gene Protein
VCP	Valosin-containing protein
XBP-1	X-box binding protein 1
